# The Duct of Luschka: An Anatomical Variant of the Biliary Tree – Two Case Reports and a Review of the Literature

**DOI:** 10.7759/cureus.14681

**Published:** 2021-04-25

**Authors:** Asmae Oulad Amar, Christine Kora, Rachid Jabi, Imane Kamaoui

**Affiliations:** 1 Radiology, Mohamed VI University Hospital, Oujda, MAR; 2 Visceral Surgery, Mohamed VI University Hospital, Oujda, MAR

**Keywords:** duct of lushka, anatomical variant, biliary tree

## Abstract

Anatomical variations occurring in the bile ducts, including the duct of Luschka, are very common. It is essential for radiologists and surgeons to understand these anomalies so they can diagnose them before and during the surgery to prevent postoperative complications. In this report, we present the case of two patients who were admitted to the emergency department for abdominal pain and fever following cholecystectomy. Postoperative imaging led to the diagnosis of injury to the duct of Luschka.

## Introduction

Anatomical variations of the bile ducts, including the duct of Luschka, are commonly encountered. Damage to these structures can lead to complications after biliary surgery, and hence they must be assessed carefully before surgeries. The incidence of ducts of Luschka varies from 12 to 50%, according to a previous study [1]. These anomalies are rarely identified before surgery and are associated with bile leakage after laparoscopic cholecystectomy.

In this report, we discuss the cases of two patients with the duct of Luschka who experienced bile leakage following cholecystectomy.

## Case presentation

Case 1

A 60-year-old man underwent laparoscopic cholecystectomy for gallstone disease. After 10 days, he was admitted to the emergency department due to right upper quadrant pain, fever, and asthenia. Physical examination revealed tenderness in the right upper quadrant.

Upon admission, the patient's complete blood count (CBC) revealed a high C-reactive protein (CRP) level (70 mg/L) and leukocyte count (12,000/mm^3^), with predominant polynuclear neutrophils. However, the liver function test (LFT) results were normal.

**Figure 1:**
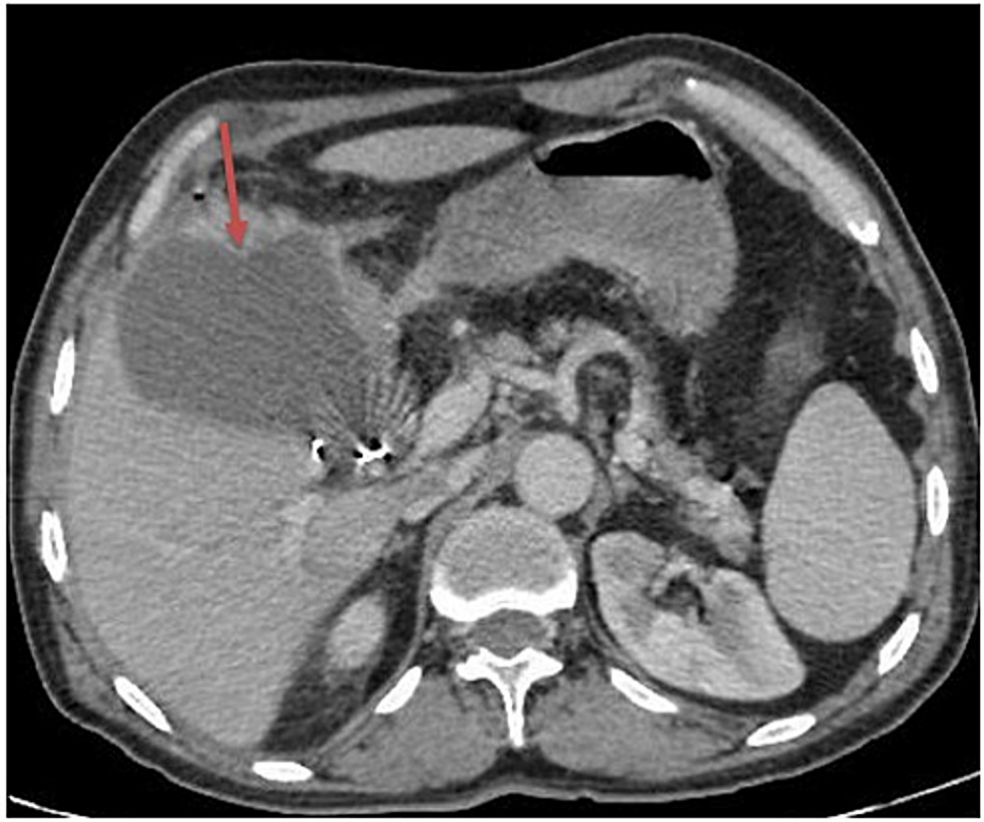
Axial contrast-enhanced CT scan The image shows vesicular bed collection with parietal enhancement (red arrow) CT: computed tomography

Abdominal ultrasonography showed a heterogenous collection (108 x 82 mm) in the vesicular bed without dilatating the bile ducts. A CT scan of the abdomen and pelvis confirmed a low-density collection in the vesicular bed, with liquid contents containing two small air bubbles and a wall enhanced after the contrast medium injection. This finding was consistent with abscess collection (Figure 1).

**Figure 2:**
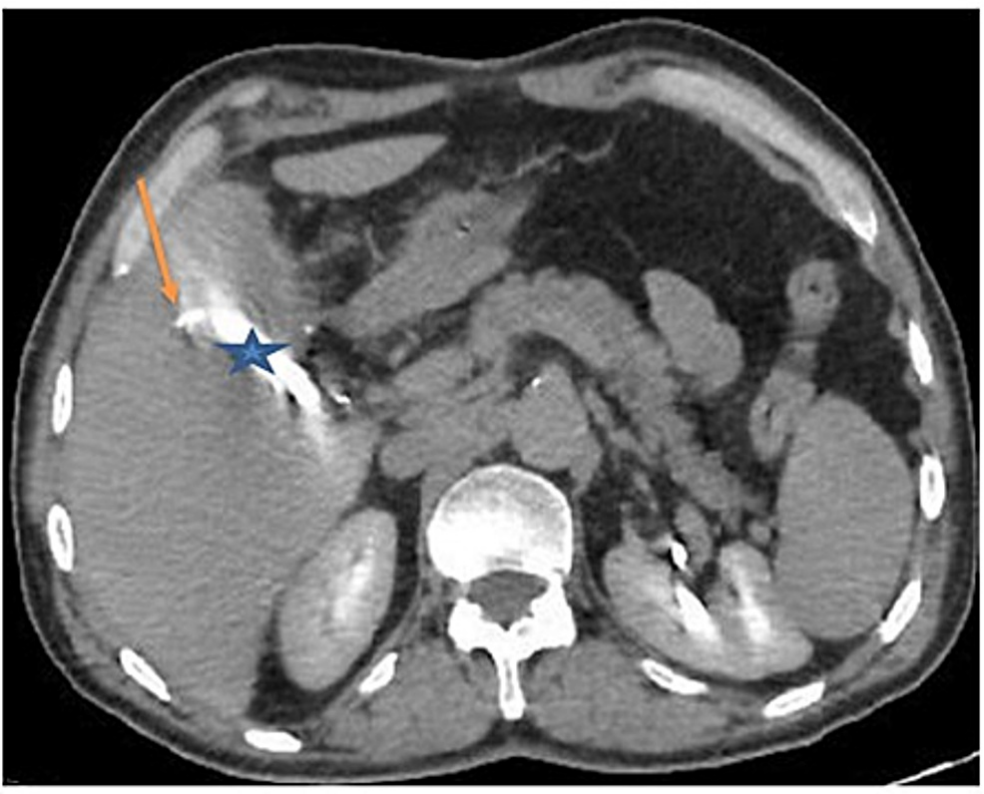
CT scan after drain opacification - view 1 The image shows opacification of the vesicular bed (blue star) and a small intrahepatic bile duct at segment V, which is connected to the injured duct of Luschka (orange arrow) CT: computed tomography

**Figure 3:**
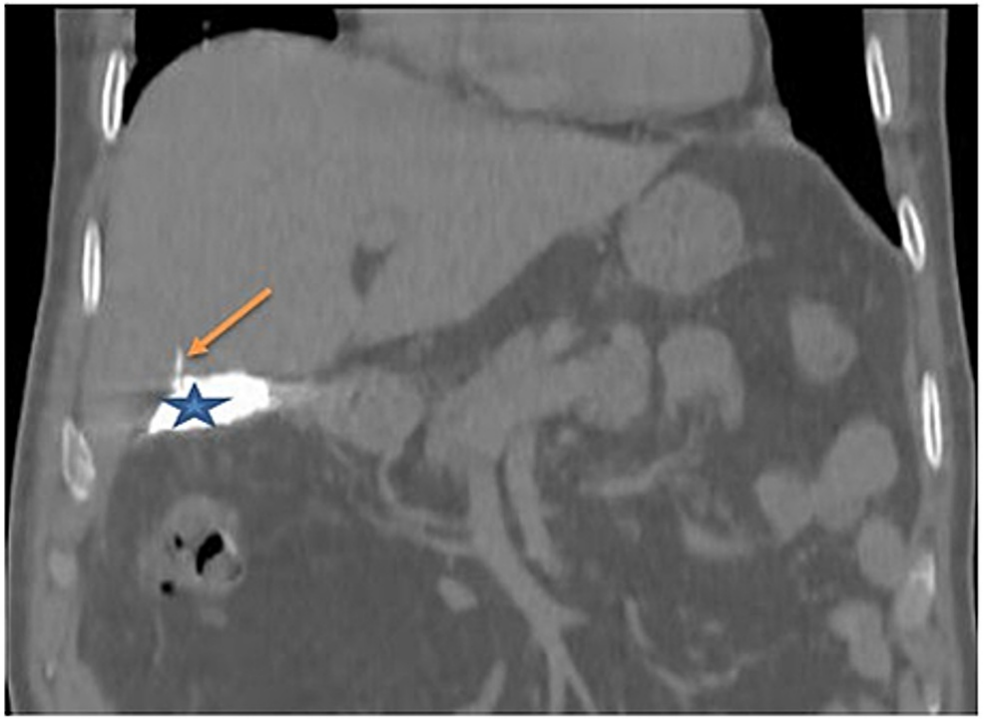
CT scan after drain opacification - view 2 The image shows opacification of the vesicular bed (blue star) and a small intrahepatic bile duct at segment V, which is connected to the injured duct of Luschka (orange arrow) CT: computed tomography

Percutaneous drainage of the collection under ultrasonography guidance was performed. Afterward, 300 cc of pus was collected. On the fifth day of admission, the drain had clear bile, with a volume of up to 200 cc/day. A cholangio-scanner (with drain opacification) revealed vesicular bed opacification and a small bile duct at segment V (Figures 2, 3). This result was characteristic of Luschka ducts. An injured Luschka duct was diagnosed during the surgery. The drain was left in place for the next six weeks, with a daily assessment of bile volume. At the end of the sixth week, the bile volume decreased from 200 to 50 cc and subsequently to 5 cc. The drain was removed after a negative clamping test, with good clinical and biological evolution.

Case 2

A 71-year-old patient was admitted to the hospital due to cholestatic jaundice with calculus acute cholecystitis and common bile duct (CBD) stones. The patient underwent a cholecystectomy with choledochotomy and stone removal with the placement of a T-tube drain and CBD suture. Preoperative cholangiography revealed bile leakage at the level of the vesicular bed. This condition was found to be caused by injury in the duct of Luschka, which was closed by separate points.

On the seventh day of postoperative care, the patient presented with fever and hepatic colic. A biological assessment revealed a high CRP level (267 mg/L) and leukocyte count (18,520/mm^3^), with predominant polynuclear neutrophils (15,650/mm^3^). Eventually, the LFT showed biochemical features of cholestatic jaundice [total bilirubin level: 12 mg/L; direct bilirubin level: 9 mg/L; gamma-glutamyl transferase (GGT) level: 243 mg/L; and alkaline phosphate level: 154 mg/L] and cytolysis [aspartate aminotransferase (AST) and alanine aminotransferase (ALT) levels that were four times higher than normal values].

**Figure 4:**
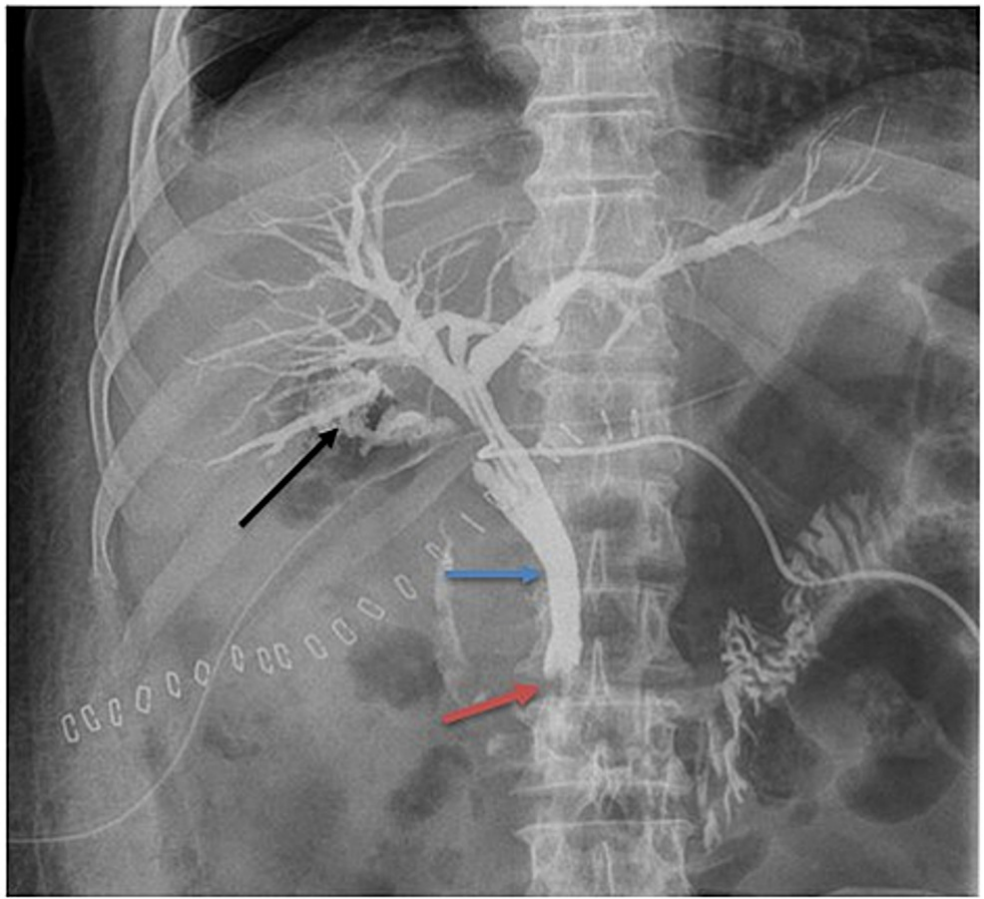
T-tube drain cholangiography The image shows dilatation of the biliary tree (blue arrow), with a cupuliform stop (red arrow) at the CBD due to residual calculus with bile leakage at the vesicular level bed (black arrow) CBD: common bile duct

**Figure 5:**
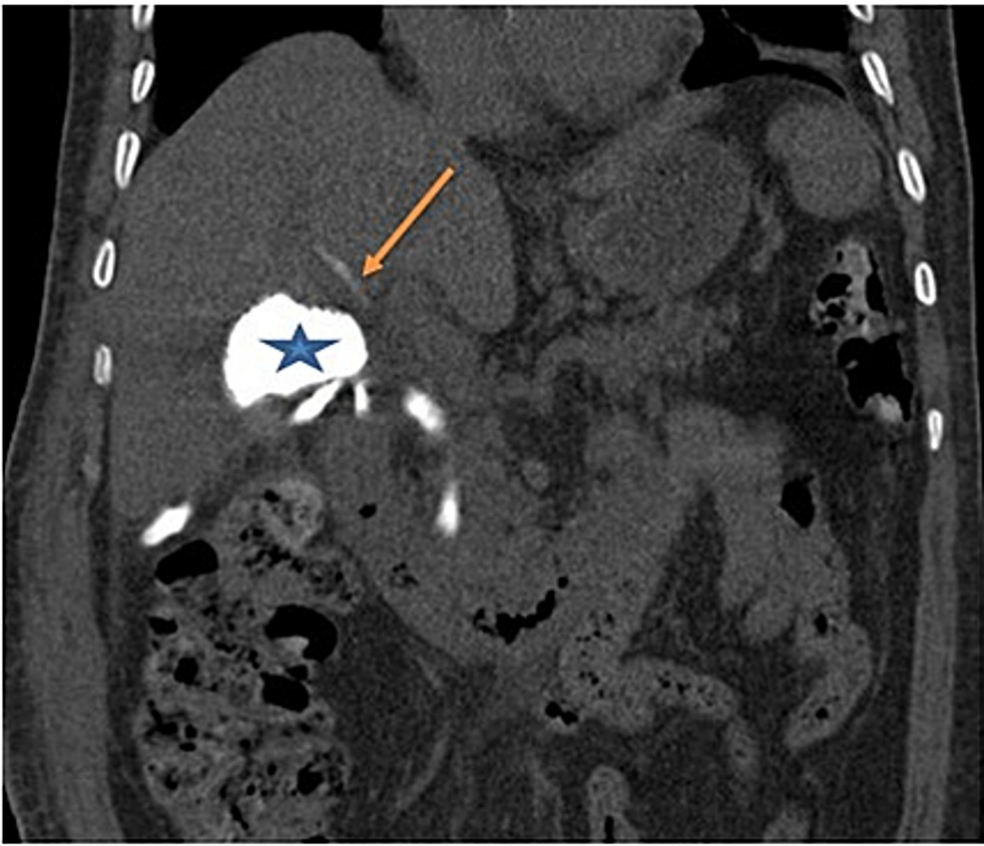
Non-contrast-enhanced CT scan after endoscopic retrograde cholangiopancreatography The image shows biliary leakage at the hilar level (blue star) with opacification of an intrahepatic bile duct at segment IV: duct of Luschka (orange arrow) CT: computed tomography

T-tube drain cholangiography revealed dilatation of the biliary tree, with a cupuliform stop at the CBD due to residual lithiasis with bile leakage at the vesicular level bed (Figure 4). Endoscopic retrograde cholangiopancreatography (ERCP) was performed to remove residual calculus. Then, a non-contrast-enhanced abdominopelvic scan objectified a leak of the contrast product injected during the ERCP in the hepatic hilum with opacification of a small pathway intrahepatic bile of segment IV related to an injury to the Luschka duct (Figure 5).

The histopathological assessment of the gallbladder revealed adenocarcinoma. The patient underwent bi-segmentectomy (IVb and V) and radical lymph node resection. Nevertheless, there were no postoperative complications.

## Discussion

Anatomical variations of the bile ducts are extremely common, and they must be assessed before biliary surgery to prevent injury. Anatomical variants of the biliary system are the secondary causes of bile leakage. They are classified into four types: the Luschka duct, also referred to as subvesicular or supravesicular duct; cystohepatic canal, also called the cholecysto-hepatic duct; segmental or sectoral bile duct variations; and aberrant bile ducts.

The Luschka ducts are the most common abnormalities, and their incidence rate varies from 12 to 50% [1]. In most cases, small bile ducts originate from the right hepatic lobe, and they do not open into the gallbladder. This differentiates them from the actual cholecystohepatic ducts. Cystohepatic ducts drain a part of the right lobe into the cystic duct or the gallbladder. Several studies have shown that the combined incidence rate of cystohepatic and cholecystohepatic canals varies from 0.2 to 2.3% [1]. Aberrant bile ducts are a rare anatomical variant comprising the bile ducts within the vesicular fossa's connective tissues.

There are two hypotheses regarding its etiology. The first involves a congenital process, occurring secondary to embryological development during the third or fourth week of gestation [2]. The second one considers it as acquired, and this notion is based on two theories. The Luschka ducts are normal peripheral ducts located in a region where the liver parenchyma has regressed due to liver remodeling, and these channels are branches of the liver parenchyma that become hypertrophic after inflammation. The prevalence of such anatomical variations is extremely low, and more detailed data are not available [2]. These bile ducts have a small diameter (about 2 mm), and they originate from the right lobe of the liver [3]. Due to their small size, they can go unnoticed and can be injured during biliary surgery.

Preoperative diagnosis of these anatomical variants is rare, with only one case report documenting a preoperative diagnosis via ultrasonography [4]. The diagnosis is generally obtained during the first week after surgery when abdominal pain, biliary peritonitis, or even sepsis [5] occurs. Further, the assessment usually reveals biliary leakage. However, there are asymptomatic patients diagnosed several weeks after surgery [5]. In our first case, the patient presented with bile leakage, and he was diagnosed based on abdominal pain secondary to an infected biloma. In the second case, the diagnosis was made during intraoperative cholangiography.

Ultrasonography is the first-line investigation modality for biliary pathologies, and it facilitates the evaluation of hepatobiliary pathology and specific anatomical variants. The Luschka ducts or cholecystohepatic canals appear as a canal with a small diameter (mm) with anechoic content at the level of the gallbladder bed (with or without continuity with the gallbladder). However, the performance of preoperative ultrasonography is limited as this technique is operator-dependent [6]. Ultrasonography is more efficient in the postoperative setting, the ultrasound objective being an outpouring located in the vesicular bed or diffuse in the abdomen; an intra or under the hepatic collection. It can also be used as a guide during puncture or drainage.

CT scan is usually a second-line investigation that can confirm ultrasonography findings by identifying postoperative complications and anatomical variants. Several techniques, particularly scanning with opacification of the drain (T-tube drain or that which evacuates the collection) can be used, as in these two cases. Percutaneous cholangiography is an invasive technique, and hence it has been less frequently used since the advent of MRI and bili-MRI with 3D sequences. Bili-MRI with 3D sequences is considered the best technique for exploring the biliary tract in anatomical variants. However, it is not systematically requested for any biliary tract pathology. In case of postoperative biliary leakage, contrast (Teslascan®)-enhanced bili-MRI is recommended.

Perioperative cholangiography is still preferred in doubtful cases. Some studies have recommended intraoperative cholangiography during laparoscopic cholecystectomy [4] for the treatment of bile leakage during the operative period itself, as in our second patient.

ERCP is the most commonly used method [7]. It can help diagnose and treat bile leakage by reducing intra-biliary pressure with endoscopic sphincterotomy and by placing a prosthesis. This method facilitates the preferential flow of the bile via the papilla, thereby allowing the healing of lesions in the subvesicular canal. Simple drainage is adequate in a few asymptomatic patients with low-volume bile leakage [2]. In our first case, a drain was placed and left in the collection until spontaneous healing of the injured bile duct occurred. Nevertheless, a second surgery can also be considered in such cases.

## Conclusions

The duct of Luschka is an extremely common anatomical variant of bile ducts. However, it is often unknown to radiologists and surgeons, and its injury can lead to many complications. Knowledge of this duct of Luschka is important so that it can be diagnosed before and during the surgery, which would help avoid its injury and associated complications.
